# Valdecoxib Protects against Cell Apoptosis Induced by Endoplasmic Reticulum Stress via the Inhibition of PERK-ATF4-CHOP Pathway in Experimental Glaucoma

**DOI:** 10.3390/ijms232112983

**Published:** 2022-10-26

**Authors:** Zhaolin Gao, Min Li, Fei Yao, Xiaobo Xia, Tianqi Duan, Jingzhuo Meng, Yanxia Huang, Ye He, Adonira Saro, Jufang Huang

**Affiliations:** 1 Department of Anatomy and Neurobiology, School of Basic Medical Science, Central South University, Changsha 410013, China; 2 Hunan Key Laboratory of Ophthalmology, Central South University, Changsha 410008, China

**Keywords:** valdecoxib, glaucoma, ER stress, apoptosis

## Abstract

The purpose of this study was to investigate the effects of valdecoxib on the retina in retinal ischemia-reperfusion injury (IRI) and R28 cells following oxygen-glucose deprivation/recovery (OGD/R) injury, as well as the underlying mechanisms. Immunofluorescence and Cell Counting Kit-8 (CCK-8) analyses were used to identify the proper timepoint and concentration of valdecoxib’s protective effect on the R28 cells in the OGD/R model. Hematoxylin-eosin (HE) staining and immunofluorescence were used to explore valdecoxib’s effect on the retina and retina ganglion cell (RGC) in IRI. Cell apoptosis was determined by a TUNEL Apoptosis Detection Kit and Annexin V-FITC/PI flow cytometry. The expression levels of p-PERK, transcription factor 4 (ATF4), GRP78, CHOP, cleaved caspase 3, bax and bcl-2 were measured by Western blot analyses. The valdecoxib protected the R28 cells from OGD/R injury by decreasing the cell apoptosis rate, and it exerted a protective effect on retinas in I/R injury by inhibiting RGC apoptosis. The valdecoxib pretreatment reversed the expression of p-PERK, ATF4, CHOP, GRP78, cleaved caspase 3 and bax induced by the glaucomatous model. Meanwhile, the CCT020312 reversed the valdecoxib’s anti-apoptosis effect by activating PERK-ATF4-CHOP pathway-mediated endoplasmic reticulum (ER) stress. These findings suggest that valdecoxib protects against glaucomatous injury by inhibiting ER stress-induced apoptosis via the inhibition of the PERK-ATF4-CHOP pathway.

## 1. Introduction

Glaucoma is the leading cause of irreversible blindness worldwide, characterized by the progressive loss of the visual field and retinal ganglion cells, as well as optic nerve damage [1]. An estimated 57.5 million people worldwide are affected by primary open angle glaucoma (POAG), with a global prevalence of 2.2% [2]. Tham et al.’s predictions include an increase in the number of people aged 40–80 years who have glaucoma from 76 million in 2020 to 111.8 million by 2040 [3]. Elevated intraocular pressure (IOP), as a significant risk factor for glaucoma, has been targeted by pharmacological or surgical therapies to slow glaucoma progression. However, a proportion of glaucoma patients still progress to blindness in spite of these IOP-controlling treatments. Therefore, it is of great significance that we develop more potential novel approaches for glaucoma treatment.

The pathogenesis of glaucoma is complicated and still under investigation, but evidence from both in vivo and in vitro models has suggested that ischemia-reperfusion injury, oxidative stress, inflammation, glutamate excitotoxicity, impaired microcirculation and dysfunctional immune responses may be involved in its onset [4,5,6,7,8]. Previous researchers have shown that, in both a chronic glaucoma model and an acute glaucoma model, an increase in endoplasmic reticulum (ER) stress proteins in the retina ganglion cells (RGCs) was observed [9,10]. The ER is affected by environmental changes, such as cellular stresses, and controls cell function/survival [11]. Different pathological and physiological conditions, such as nutrient scarcity, changes in redox status and viral infection, can influence the ER’s ability to facilitate protein folding, potentially resulting in unfolded or misfolded protein accumulation in the ER lumen and, consequently, increased ER stress. Several studies have suggested that ER stress is associated with neuronal cell death in neurodegenerative diseases such as glaucoma [9,12,13]. When using medicine that blocks ER stress, damage can be reduced and the survival rate of RGC improved [14]. CHOP knockout (KO) mice, which act as the common ER stress blockage model, demonstrated increased RGC survival by 24% after two weeks of ON axotomy [15]. These studies demonstrated that ER stress plays an important role in the pathological process of glaucoma. Finding new therapies that target ER stress has been proposed to be effective for glaucoma treatment. 

Valdecoxib is a selective COX-2 inhibitor, which has been widely used in clinical practice for the treatment of osteoarthritis (OA) of the knees and hips [16,17,18], rheumatoid arthritis [18], analgesia in dysmenorrhea [19] and postoperative analgesia after hip arthroplasty [20], orthopedic foot and oral surgery [21,22]. Kim et al. found that valdecoxib improves lipid-induced skeletal muscle insulin resistance via the simultaneous suppression of inflammation and endoplasmic reticulum stress, suggesting that valdecoxib is relevant to ER stress under certain conditions [23]. However, the question of whether valdecoxib has an effect on ER stress in glaucomatous injury has not been studied.

In this study, we investigated valdecoxib’s effects on glaucomatous damage using an I/R rat model and an OGD/R cell model to elucidate the underlying molecular mechanisms.

## 2. Results

### 2.1. Valdecoxib Protects R28 from OGD/R Injury by Inhibiting Apoptosis In Vitro

**Figure 1:**
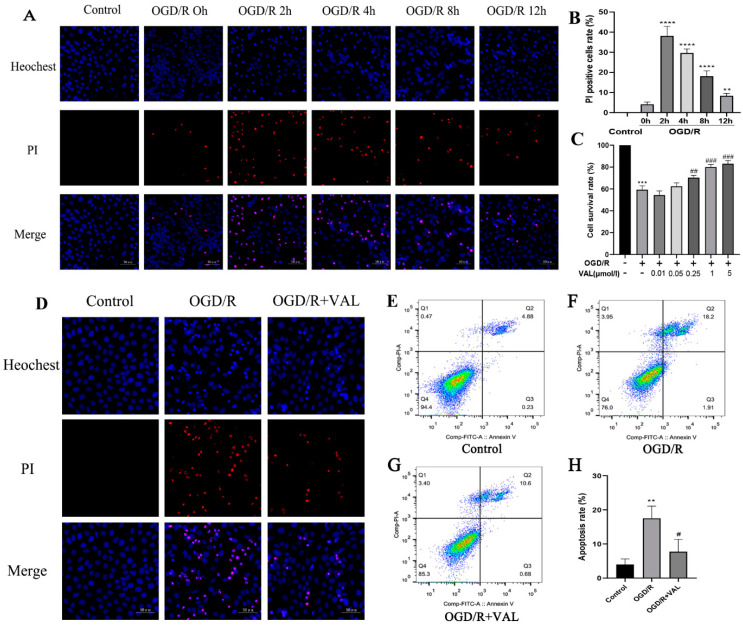
Valdecoxib reduces the number of PI-positive R28 cells and the cell apoptosis rate in the OGD/R model. R28 cells were pretreated with the indicated concentrations of valdecoxib for 4 h. (**A**) R28 cells in the control group and at multiple time points for the OGD/R model groups, stained with PI (red) and Hoechest (blue). Scale bar = 50 μm. (**B**) Proportion of PI-positive R28 cells in the control group and OGD/R 0 h, 2 h, 4 h, 8 h and 12 h groups. (**C**) CCK-8 was used to detect R28 cells’ survival rates in the control group, OGD/R group and OGD/R groups pretreated with valdecoxib of different concentrations. The data show a significant increase in the cell survival rate observed at the concentrations of 1 and 5 μm VAL compared to the OGD/R group. (**D**) R28 cells in the control, OGD/R and OGD/R+VAL groups, stained with PI (red) and Hoechest (blue). Scale bar = 50 μm. (**E**–**H**) Valdecoxib reduced the cell apoptosis rate in the OGD/R model as measured by annexin V-FITC/PI staining and flow cytometry analysis. Q3: early apoptosis, Q2: late apoptosis. The untreated control group is assigned a survival rate of 100%. Data are presented as the mean ± SD of three independent experiments. **** *p* < 0.0001, ** *p* < 0.01 vs. control group in Figure 1B. *** *p* < 0.001 vs. control group, ^##^ *p* < 0.01, ^###^ *p* < 0.001 vs. OGD/R group in Figure 1C. ** *p* < 0.01 vs. control group, ^#^ *p* < 0.05 vs. OGD/R group in Figure 1H.

As a first step to explore OGD/R-mediated cell death, we detected the cell death rate at multiple time points with the OGD/R model using PI staining. PI-positive cells were identified as dead cells. The proportions of PI-positive cells at multiple time points were calculated. The highest PI-positive cell rate was observed at 2 h after OGD/R (Figure 1A,B). Next, to test whether the valdecoxib could protect the R28 from OGD/R-mediated cell death, CCK-8 was performed to identify the valdecoxib’s effects on the R28 cells in the OGD/R model at different concentrations at 2 h post-OGD/R. The valdecoxib treatment significantly elevated the cell survival rate at the concentrations of 1 and 5 μmol/L (Figure 1C). The former was chosen to detect the valdecoxib’s protective effect on the R28 cells in the OGD/R model in the subsequent experiments. No significant cell death was observed in control groups pretreated with different concentrations of valdecoxib (Supplementary Figure S1). PI staining was further used to determine the valdecoxib’s protective effect (Figure 1D). To identify whether the valdecoxib’s effect involved an anti-apoptosis mechanism, flow cytometry was performed, and a further analysis indicated both that OGD/R induced cell apoptosis and the valdecoxib decreased the cell apoptosis rate (Figure 1E–H). Based on these results, we conclude that valdecoxib protects R28 cells from OGD/R injury by inhibiting apoptosis.

### 2.2. Valdecoxib Protects the Retina from Ischemia-Reperfusion Injury (IRI) by Inhibiting Apoptosis In Vivo

**Figure 2:**
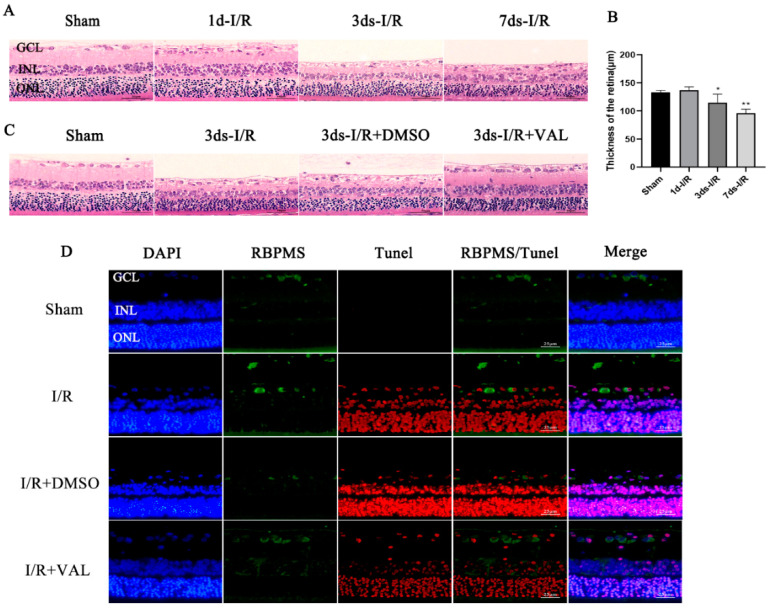
Valdecoxib alleviates the morphological changes to the retina and reduces the number of TUNEL-positive RGCs in retinal ischemia-reperfusion injury. (**A**) Representative images of vertical sections obtained from retinas in the control, 1d-I/R, 3ds-I/R and 7ds-I/R groups, stained with hematoxylin (blue) and eosin (red). Scale bar = 50 μm. (**B**) Quantification of the mean total thickness of the retina in the control and 1-, 3- and 7-day post-I/R groups. The retinas of the 3ds-I/R and 7ds-I/R groups were significantly thinner compared to those of the control group. (**C**) Representative images of vertical sections obtained from retinas in the control, 3ds-I/R, 3ds-I/R+DMSO and 3ds-I/R+VAL groups, stained with hematoxylin (blue) and eosin (red). Scale bar = 50 μm. (**D**) Images obtained from retinas in the control, I/R, I/R+DMSO and I/R+VAL groups, stained with DAPI (blue), RBPMS (green) and TUNEL (red). Scale bar = 25 μm. RBPMS was used to label RGCs, and TUNEL was used to label apoptotic cells. The data are presented as the mean ± SD of three independent experiments. Each group was composed of five rats. ** *p* < 0.01, * *p* < 0.05 vs. sham group.

Eight-week-old SD rats were sacrificed and their eyeballs were removed at 1, 3 or 7 days post-IRI. HE staining was performed to detect the morphological changes in the retina. We discovered that, compared to the control group, the retinas from 3 and 7 days post-IRI were markedly thinner. Additionally, lost RCGs or their disordered arrangement was observed at 3 and 7 days after IRI when compared with the control retina (Figure 2A,B). We next determined the valdecoxib’s effect on the RGCs in the I/R model by HE staining and immunofluorescence. The valdecoxib significantly increased the retinal thickness and RGCs’ survival rate at 3 days post-injury (Figure 2C). A TUNEL assay was performed to clarify whether valdecoxib’s protective effect involves the anti-apoptosis mechanism. RBPMS and TUNEL were used to label the RGCs and apoptotic cells of the retina, respectively. We demonstrated that the TUNEL-positive RGCs increased after I/R and were reduced after the valdecoxib treatment compared to the I/R group (Figure 2D). The above results indicate that valdecoxib can attenuate IRI-induced RGC loss and retina injury by inhibiting apoptosis.

### 2.3. Valdecoxib Inhibits R28 Apoptosis by Alleviating PERK-ATF4-CHOP Pathway-Mediated ER Stress

**Figure 3:**
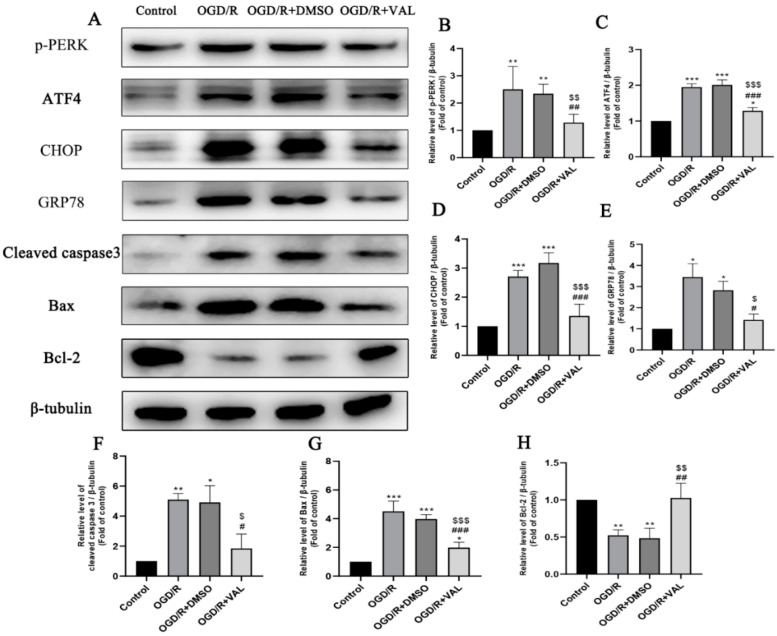
Effect of valdecoxib on the expression of ER stress-related and apoptosis-related proteins in the OGD/R model. (**A**) Western blot analysis of p-PERK, ATF4, GRP78, CHOP, cleaved caspase 3, bax and bcl-2 levels in the control, OGD/R, OGD/R+DMSO and OGD/R+VAL groups. (**B**–**H**) Quantification of expression levels of p-PERK, ATF4, GRP78, CHOP, cleaved caspase 3, bax and bcl-2 in the control, OGD/R, OGD/R+DMSO and OGD/R+VAL groups using the densitometric analyses of Western blotting. The bar charts show the quantitative data (normalized by β-tubulin) for each protein relative to the control group (assigned a value of 1). Data are represented as the mean ± SD of three independent experiments. One-way ANOVA is used in B to H. * *p* < 0.05, ** *p* < 0.01, *** *p* < 0.001 vs. control group. ^#^ *p* < 0.05, ^##^ *p* < 0.01, ^###^ *p* < 0.001 vs. OGD/R group. ^$^ *p* < 0.05, ^$$^ *p* < 0.01, ^$$$^ *p* < 0.001 vs. OGD/R+DMSO group.

Previous studies showed that OGD/R induced ER stress and increased activating transcription factor-4 (ATF4) and CHOP protein levels. We further examined whether the protein kinase RNA-like endoplasmic reticulum kinase (PERK)-ATF4-CHOP pathway was inhibited in the OGD/R model after valdecoxib pretreatment. Western blotting (Figure 3A) and its densitometric analyses (Figure 3B–H) demonstrated that the GRP78, p-PERK, CHOP and ATF4 protein levels were markedly upregulated in the OGD/R and OGD/R+DMSO groups compared to the control group. The valdecoxib decreased the expression of those proteins during OGD/R injury. In addition, compared to the control group, the OGD/R and OGD/R+DMSO groups significantly elevated the apoptosis-related proteins, including bax and cleaved caspase 3, while these proteins were decreased in the valdecoxib pretreatment group (Figure 3A). The expression of the anti-apoptosis protein bcl-2 was the opposite to that of the bax and cleaved caspase 3 in each group. The expression levels of the pro-apoptosis proteins bax and cleaved caspase3 were upregulated, along with the activation of the PERK-ATF4-CHOP pathway, and decreased together with the inhibition of the PERK-ATF4-CHOP pathway. These data demonstrate that valdecoxib may inhibit R28 cells’ apoptosis by alleviating PERK-ATF4-CHOP pathway-mediated ER stress.

### 2.4. Valdecoxib Protects the Retina from Ischemia Reperfusion Injury (IRI)-Mediated Apoptosis by Alleviating PERK-ATF4-CHOP Pathway-Mediated ER Stress

**Figure 4:**
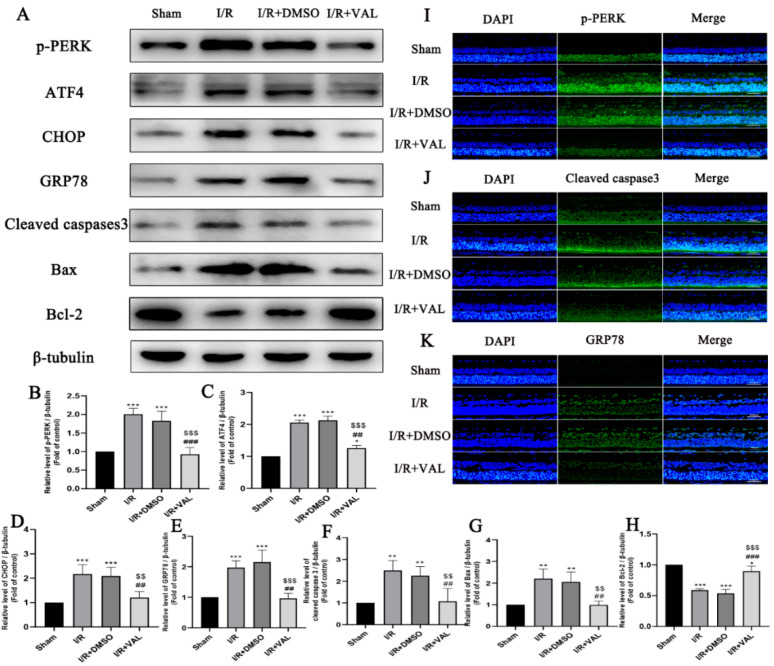
Effect of valdecoxib on the expression of ER stress-related and apoptosis-related proteins in the retinal ischemia-reperfusion model. (**A**) Western blot analysis of p-PERK, ATF4, GRP78, CHOP, cleaved caspase 3, bax and bcl-2 levels in the control, I/R, I/R+DMSO and I/R+VAL groups. β-tubulin served as the loading control. (**B**–**H**) Quantification of expression levels of p-PERK, ATF4, GRP78, CHOP, cleaved caspase 3, bax and bcl-2 in the control, I/R, I/R+DMSO and I/R+VAL groups using the densitometric analyses of Western blotting. The bar charts show the quantitative data (normalized by β-tubulin) for each protein relative to the sham group (assigned a value of 1). (**I**) Representative fluorescence images of p-PERK staining are shown (scale bar = 50 μm). Immunostaining was executed using a primary antibody against p-PERK (green), and the nucleus (blue) is marked by DAPI. (**J**) Representative fluorescence images of cleaved caspase 3 staining are shown (scale bar = 50 μm). Immunostaining was executed using a primary antibody against cleaved caspase 3 (green), and the nucleus (blue) is marked by DAPI. (**K**) Representative fluorescence images of GRP78 staining are shown (scale bar = 50 μm). Immunostaining was executed using a primary antibody against GRP78 (green), and the nucleus (blue) is marked by DAPI. Images demonstrate the increased expression level of p-PERK, cleaved caspase 3 and GRP78 in the I/R and I/R+DMSO groups, and decreased expression level of those proteins in the I/R+VAL group in the retinal ganglion cell layer. Data are represented as the mean ± SD of three independent experiments. Each group was composed of five rats. One-way ANOVA is used in B to H. * *p* < 0.05, ** *p* < 0.01, *** *p* < 0.001 vs. sham group. ^##^ *p* < 0.01, ^###^ *p* < 0.001 vs. I/R group. ^$$^ *p* < 0.01, ^$$$^ *p* < 0.001 vs. I/R+DMSO group.

After the valdecoxib’s effect on the PERK-ATF4-CHOP pathway and the cell apoptosis in the OGD/R model were demonstrated, we examined whether valdecoxib exerts a protective effect on the retina in IRI through similar mechanisms. Retina lysates were subjected to Western blot analysis, which demonstrated that the expression levels of p-PERK, ATF4 and CHOP increased in the I/R and I/R+DMSO groups compared to the control retina, while these proteins were reduced in the retinas pretreated with valdecoxib during the I/R. The expression of the ER stress protein GRP78 was consistent with the PERK-ATF4-CHOP pathway proteins (Figure 4A–H). The expression levels of the apoptosis-related proteins were examined in the control, I/R, I/R+DMSO and I/R+valdecoxib groups. As shown in Figure 4A, when using Western blotting, compared to the control, there was an increased expression of bax and cleaved caspase 3 in the retinas of the I/R and I/R+DMSO groups. The valdecoxib decreased the expression of these proteins in the retina during I/R injury. The expression of the anti-apoptosis protein bcl-2 was the opposite of that of the bax and cleaved caspase 3 in each group (Figure 4A–H). The expression levels of p-PERK, cleaved caspase 3 and GRP78 in RGCs were evaluated by immunofluorescence. The results obtained in the immunofluorescence analyses were in accordance with the corresponding Western blot results (Figure 4I–K). These results support the conclusion that valdecoxib protects the retina from IRI-mediated apoptosis by alleviating PERK-ATF4-CHOP pathway-mediated ER stress.

### 2.5. CCT020312 Reverses Valdecoxib’s Anti-Apoptosis Effect by Activating PERK-ATF4-CHOP Pathway-Mediated ER Stress In Vitro 

**Figure 5:**
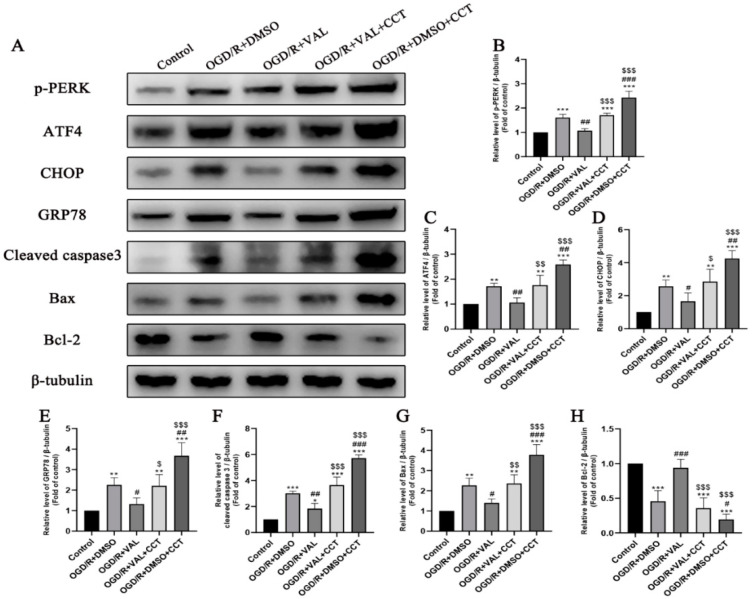
PERK activator CCT020312 reverses the effect of valdecoxib on the expression of ER stress-related proteins and on the apoptosis-related proteins in the OGD/R model. R28 cells were pretreated with CCT020312 at the concentration of 5 μmol/L for 4 h. (**A**) Western blot analysis of p-PERK, ATF4, GRP78, CHOP, cleaved caspase 3, bax and bcl-2 levels in the control, OGD/R+DMSO, OGD/R+VAL, OGD/R+VAL+CCT and OGD/R+DMSO+CCT groups. β-tubulin served as the loading control. (**B**–**H**) Quantification of expression levels of p-PERK, ATF4, GRP78, CHOP, cleaved caspase 3, bax and bcl-2 in the control, OGD/R+DMSO, OGD/R+VAL, OGD/R+VAL+CCT and OGD/R+DMSO+CCT groups using the densitometric analyses of Western blotting. The bar charts show the quantitative data (normalized by β-tubulin) for each protein relative to the control group (assigned a value of 1). Data are represented as the mean ± SD of three independent experiments. One-way ANOVA is used in B to H. * *p* < 0.05, ** *p* < 0.01, *** *p* < 0.001 vs. control group. ^#^ *p* < 0.05, ^##^ *p* < 0.01, ^###^ *p* < 0.001 vs. OGD/R+DMSO group. ^$^ *p* < 0.05, ^$$^ *p* < 0.01, ^$$$^ *p* < 0.001 vs. OGD/R+VAL group.

Recent studies have identified that CCT020312, as a selective activator of PERK, can activate the PERK-ATF4-CHOP signaling pathway [24]. We examined whether activating the PERK-ATF4-CHOP pathway by using CCT020312 can reverse the anti-apoptosis effect of valdecoxib in the OGD/R model. The R28 cells were pretreated with different concentrations of CCT020312 and no significant cell death was observed. The CCT020312 activated p-PERK at the concentrations of 3 and 5 μmol/L (Supplementary Figure S2). The latter was chosen to detect the CCT020312’s effect on the R28 cells in the OGD/R model in the subsequent experiments. The R28 was pretreated with valdecoxib prior to CCT020312 administration, after which it was subjected to the OGD/R model. The cell lysates collected from each group were subjected to a Western blot analysis of various markers of ER stress, apoptosis and the PERK-ATF4-CHOP pathway. A densitometric analysis confirmed that the valdecoxib significantly reduced the OGD/R-induced GRP78, p-PERK, ATF4 and CHOP, as well as the expression of apoptosis-related proteins, including bax and cleaved caspase 3. The CCT020312 reversed the valdecoxib’s effects, increasing the expression of markers of ER stress and the PERK-ATF4-CHOP pathway (Figure 5A–H). We next determined whether the activation of the PERK-ATF4–CHOP pathway induced by the CCT020312 increased the expression of the apoptosis-related proteins. The Western blot results showed that the expression levels of the pro-apoptosis proteins, including bax and cleaved caspase 3, were consistent with those of the PERK-ATF4-CHOP pathway proteins in the CCT020312 pre-treated group (Figure 5A–H). These studies support the hypothesis that CCT020312 reverses valdecoxib’s anti-apoptosis effect by activating PERK-ATF4-CHOP pathway-mediated ER stress.

## 3. Discussion

A wide variety of mechanisms are involved in the development and progression of glaucoma. ER stress has been recognized as playing a significant role in the pathology of glaucoma [12]. Valdecoxib’s protective effect has been implicated in several neurodegenerative diseases; however, its role in glaucoma is largely unknown [25,26,27]. In this context, our study investigated whether valdecoxib can perform a rescue function in glaucomatous models, and whether the underlying molecular mechanisms are involved in ER stress. 

We explored valdecoxib’s protective effect against glaucomatous models in vivo and in vitro. We found that pretreatment with valdecoxib reversed the morphological changes of RGC loss and thinner retinas caused by IRI. In addition, the expression of pro-apoptosis proteins increased in the I/R group and decreased after valdecoxib treatment. These results suggest that valdecoxib can alleviate I/R-induced glaucoma-like damage through the inhibition of apoptosis in RGCs. Similar results were observed in a cell model of glaucoma, demonstrating the key role of apoptosis in glaucoma pathology. This is consistent with other studies showing that the apoptosis of RGCs is the final common pathway in both human and experimental models of glaucoma [28,29,30,31]. Inhibiting apoptosis has proven to be effective for glaucoma protection [32,33]. In our study, the anti-apoptosis effect of valdecoxib was revealed in the glaucomatous models. Therefore, we speculate that this may be the possible mechanism through which valdecoxib confers protection against glaucoma. However, previous studies suggested that valdecoxib has a pro-apoptosis effect in tumor cell lines [34,35]. Cyclooxygenase-2 (COX-2) is a gene encoding an inducible prostaglandin synthase enzyme that is overexpressed in many tumors [36]. A study demonstrated that downregulating COX-2 induced cell apoptosis in hepatocellular carcinoma [37]. Therefore, we can infer that COX-2 may be the target for valdecoxib to induce cell apoptosis in tumors. In addition, other studies indicated that the effect of valdercoxib on apoptotic cell death is cell-line-dependent [35]. Our study revealed that valdercoxib exerts an anti-apoptosis effect in glaucomatous models. This may be for two reasons. First, valdercoxib may respond differently to various cell lines. It exerts pro-apoptosis effects on tumor cell lines, but may inhibit apoptosis in other cell lines. Moreover, we speculate that valdercoxib’s anti-apoptosis effect may be induced via COX-2 independent mechanisms in glaucomatous models. This is the first study to identify valdecoxib’s anti-apoptosis mechanism in glaucoma protection.

We further explored the underlying molecular mechanism of valdecoxib’s anti-apoptosis effect in glaucomatous models. ER stress-induced apoptosis has been implicated in the pathogenesis of various diseases [38]. Inhibiting ER stress can protect cells against apoptosis [39,40]. Studies have revealed that increasing ER stress seems to be one reason for IOP and the development of glaucoma [41]. We speculate that ER stress may be the target of valdecoxib to suppress apoptosis in glaucomatous models. PERK-ATF4-CHOP is one of the classical pathways of ER stress and is pro-apoptosis [42]. When ER stress is prolonged, the activation of PERK can promote the translation of ATF4, which increases the transcription of specific unfolded protein response (UPR) target genes including CHOP [43]. In this study, we detected the expression of the ER stress protein in each group. We showed that the valdecoxib suppressed the expression of p-PERK, ATF4, CHOP and GRP78 induced by I/R injury. The apoptosis-related protein levels, including bax, bcl-2 and cleaved caspase 3, were further detected. The results showed that the activation of the PERK-ATF4-CHOP pathway positively altered the pro-apoptosis proteins’ expression levels and negatively altered the anti-apoptosis proteins. The pretreatment with valdecoxib inhibited the activation of the PERK-ATF4-CHOP pathway and improved the expression level of the anti-apoptosis protein. Similar results were obtained in the cell model of glaucoma. These results indicated that valdecoxib attenuated ER stress-mediated apoptosis via the inhibition of the PERK-ATF4-CHOP pathway. Our study revealed that PERK-ATF4-CHOP can offer a potential target for glaucoma treatment. In addition, CCT020312 administration abolished valdecoxib’s protective effect, activated the expression of p-PERK, ATF4 and CHOP and aggravated ER stress-mediated apoptosis in the OGD/R model, indicating that the inhibition of the PERK-ATF4-CHOP pathway was required for valdecoxib’s protective effect on glaucoma.

This is the first study to explore valdecoxib’s function in glaucomatous models, providing a potential treatment for glaucoma. In addition, our results provide clues as to how we can better understand the mechanism of ER stress in glaucoma. However, while the experiments were performed in vivo and in vitro to detect valdecoxib’s function in glaucoma models, there were limitations to our study. We used the acute intraocular pressure elevation model (aHIOP) to mimic retinal I/R injury. Although this is one of the classic glaucoma models and it was chosen for a number of glaucoma research studies, the results we obtained in the I/R model have not yet been tested on other glaucoma models in vivo. It is uncertain whether the same results can be obtained in other glaucoma animal models. More glaucoma models need to be considered to test our outcomes.

In conclusion, our study indicates that the PERK-ATF4-CHOP pathway plays a significant pathological role in glaucomatous damage. Valdecoxib protects against glaucomatous injury by inhibiting endoplasmic reticulum stress-induced apoptosis via the inhibition of the PERK-ATF4-CHOP pathway. These findings suggest a promising role for valdecoxib therapy in protecting individuals from glaucoma.

## 4. Materials and Methods

### 4.1. Cell Culture

R28 retinal neuronal-like cells were obtained from Dr. Kun Xiong (Department of Anatomy and Neurobiology, School of Basic Medical Sciences, Central South University, Changsha, China) and cultured in low-glucose DMEM with 10% fetal bovine serum (FBS; Cat#SH30256.01, Thermo Scientific, Carlsbad, CA, USA) and 1% penicillin–streptomycin (PS; Cat#SV30010, Thermo Scientific) at 37 °C in a humidified atmosphere containing 5% CO_2._

### 4.2. Animals

The rats used in the retinal I/R model were 8-week-old male Sprague–Dawley rats purchased from Slaccas, Changsha, China and bred in animal facilities of Central South University (Changsha, China). The study protocols were approved by the Animal Research Committee of the Xiangya School of Medicine (approval number: CSU-2022-0088, date of approval: 22 February 2022). All animal experiments were carried out in accordance with the Guide for the Care and Use of Laboratory Animals (National Institutes of Health, Bethesda, MD, USA). 

### 4.3. Reagents and Antibodies

We purchased propidium iodide (Cat: #81845, Sigma, St. Louis, MO, USA); Cell Counting Kit-8 (Cat: C0037, Beyotime, Shanghai, China), Annexin V-FITC/PI Kit (Cat: MA0220, Meilunbio, Dalian, China), TUNEL BrightRed Apoptosis Detection Kit (Cat: A113-03, Vazyme Biotech, Nanjing, China), agonist: CCT020312 (Cat: # HY-119240, MedChemExpress, Monmouth Junction, NJ, USA), primary antibody: RBPMS (Cat: 1830-RBPMS, PhosphoSolutions, Aurora, CO, USA, 1:200), GRP78 antibody (Cat: R24509, Zenbio, Chengdu, China, 1:1000), CHOP antibody (Cat: 1120880, Wanleibio, Shenyang, China, 1:1000), ATF4 (Cat: 381426, Zenbio, Chengdu, China, 1:500), PERK (phospho Thr981; Cat: YP1055, ImmunoWay Biotechnology Company, Plano, USA, 1:1000), bax (Cat: 50599-2-Ig, Proteintech, Wuhan, China, 1:2000), bcl-2 (Cat: 12789-1-AP, Proteintech, Wuhan, China, 1:1000) and caspase 3 (Cat: 19677-1-AP, Proteintech, Wuhan, China, 1:500).

### 4.4. I/R Injury Model (IRI) 

We used the protocol described in our team’s previous work to build the rat I/R model [44]. I/R injury was induced by elevating the intraocular pressure. Briefly, we anesthetized rats with 2% sodium pentobarbital by intraperitoneal injection. Rats were fixed on a stereotaxic instrument after using eye drops comprising obucaine, levofloxacin and compound tropicamide on the cornea. Next, a 31-G needle was inserted into the anterior chamber of the rat’s eye, and the other side of the needle was connected to normal saline by an infusion set tube. The intraocular pressure was increased to 110 mmHg gradually for 1 hour. Next, we removed the needle from the anterior chamber. Tobramycin dexamethasone eye ointment was used after surgery to prevent eye infections.

#### Valdecoxib Treatment in I/R Model

In vivo, an anesthetized rat with dilated pupils was put under a stereomicroscope, and 3 μL of 5 μM valdecoxib was injected into the vitreous chamber using a 5-μL Hamilton syringe (Hamilton AG, Bonaduz, Switzerland) 30 min before the I/R model. Four concentrations (1, 5, 25 and 100 μM) were initially chosen based on the concentration of valdecoxib we used in the OGD/R cell model and the volume of the vitreous chamber, and were confirmed empirically; 5 μM was identified as the proper concentration of valdecoxib in the glaucomatous animal model (Figure S3).

### 4.5. R28 Cell OGD/R Injury Model

For the R28 cells used to build the OGD/R model, we replaced the low-glucose DMEM with free-glucose DMEM and kept the cells under hypoxic conditions in a closed container at 37 °C for 2 h. Next, we replaced the free-glucose DMEM with low-glucose DMEM and returned the cells to the culture incubator at 37 °C and 5% CO_2_. 

### 4.6. Hematoxylin and Eosin (HE) Staining

The morphological changes to the retina induced by I/R were visualized through HE staining. We removed the rats’ eyeballs in each group and placed them in 4% paraformaldehyde for fixation, for 48 h. The eye tissue was embedded in paraffin, and 4 μm-thick slices were prepared. We followed the protocol for HE staining that was described previously [45].

### 4.7. Terminal Deoxynucleotidyl Transferase-Mediated Nick End-Labeling (TUNEL)

The eye tissue was embedded in paraffin and 4 μm-thick slices were prepared. We used a TUNEL BrightRed Apoptosis Detection Kit to detect the apoptosis RGCs of retinal tissue. The TUNEL positive cells’ nuclei were stained red. The slides were stained with DAPI to detect all cells’ nuclei.

### 4.8. Cell Counting Kit-8 (CCK-8)

The Cell Counting Kit-8 assay kit was used to detect the cell viability. A microplate reader was used to detect the absorbance values at 400 nm after the cells were incubated with CCK-8 for 2 h. The cell survival rate was then calculated. 

### 4.9. Western Blot Assay

Western blotting was performed following the routine protocol. Briefly, cells and retinal tissue were obtained after treatment under the indicated conditions. The cells and retina were harvested and mixed with lysate in tubes. Then, the tubes were placed on ice for 30 min. The supernatant of the total lysates was collected after centrifugation (12,000× *g*, 20 min, 4 °C). The protein concentration was detected using a commercial BCA Protein Assay Kit (CWBIO, Beijing, China). Next, 30-μg of protein was loaded and separated by 10% SDS-PAGE, then transferred to a nitrocellulose (NC) membrane. After blocking with 5% bovine serum albumin (BSA) at room temperature for 2 h, the membranes were incubated with the primary antibody at 4 °C overnight. The membranes were washed three times, each for 10 min. The membranes were then incubated with the second antibody for 1 h at room temperature. The membranes were washed and the band was visualized using the imaging system and quantified by densitometry using ImageJ software.

### 4.10. Annexin V-FITC/PI Flow Cytometry

A flow cytometry assay was used for the detection of cell apoptosis. Cells were made into single-cell suspensions and stained with Annexin V-FITC and PI according to the product’s instructions, after treatment with the indicated conditions. The results were quantified and analyzed using a flow cytometer.

### 4.11. Immunofluorescence Staining Assay

The immunofluorescence staining process followed the routine protocol. Briefly, first, paraffin-embedded sections were deparaffinized using xylene, followed by rehydration in serial alcohol dilutions. Next, antigen retrieval with citrate buffer, permeabilization and blocking were performed. Subsequently, the sections were incubated with the primary antibody at 4 °C overnight. Next, they were washed three times, each for 10 min. The samples were incubated with the appropriate secondary antibody (goat anti-rabbit AlexaFluor^®®^ 488, ab150077, Abcam, Cambridge, MA, 1:100; goat anti-guinea pig Alexa Fluor^®®^ 488, ab150185, Abcam, Cambridge, MA, 1:100) for 2 h. The samples were washed three times with PBS and counterstained with 1X PureBlu DAPI (BioRad, Hercules, CA, USA) for 5 min, then mounted. The slides were then viewed using a fluorescence microscope [46].

### 4.12. Propidium Iodide (PI) Staining

PI was used to detect the cell permeability. Dead cells’ nuclei stained red while the normal cells were PI-negative. We applied the protocol described in our team’s previous work [44]. The numbers of PI-positive (red) cells and Hoechst-positive (blue) cells were counted using ImageProPlus software, and the proportion of PI-positive (red) cells was calculated.

### 4.13. Statistical Analysis

All the data were presented as the mean ± standard deviation (MD). SPSS 22.0 software was used to analyze all the data, and statistical comparisons were performed using one-way ANOVA. *p*-values of <0.05 between datasets were considered statistically significant.

## Data Availability

The data presented in this study are available on request from the corresponding author.

## Supplementary Materials

The following supporting information can be downloaded at: https://www.mdpi.com/article/10.3390/ijms232112983/s1.
