# Supplementary figures and images for: Valdecoxib Protects against Cell Apoptosis Induced by Endoplasmic Reticulum Stress via the Inhibition of PERK-ATF4-CHOP Pathway in Experimental Glaucoma
Source: Int J Mol Sci. 2022 Oct 26;23(21):12983. doi: 10.3390/ijms232112983 (PMC9657191; doi:10.3390/ijms232112983)

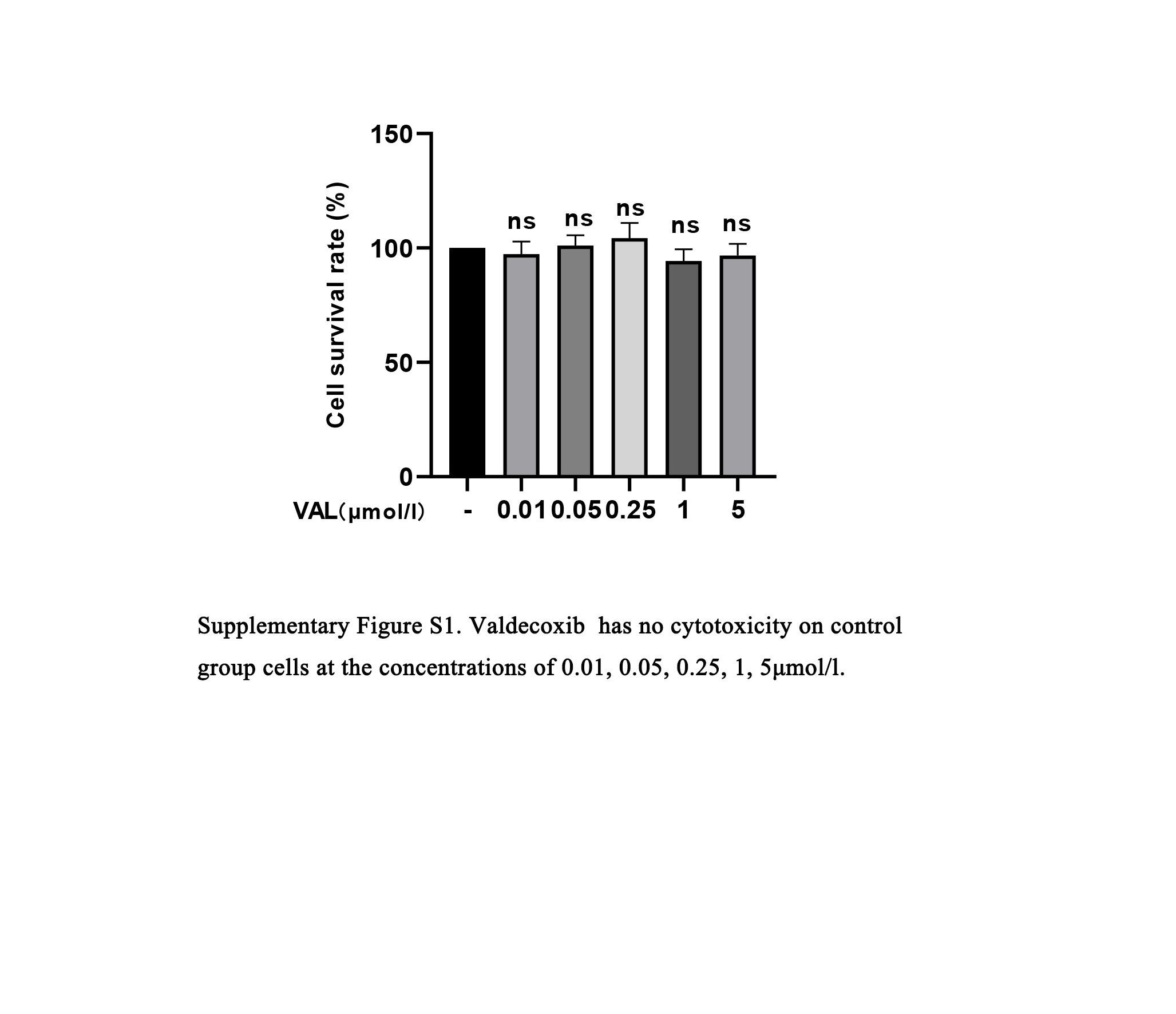

Supplement: Supplementary file 1 [file ijms-23-12983-s001.zip › Supplementary materials/Supplementary Figure S1.tif]

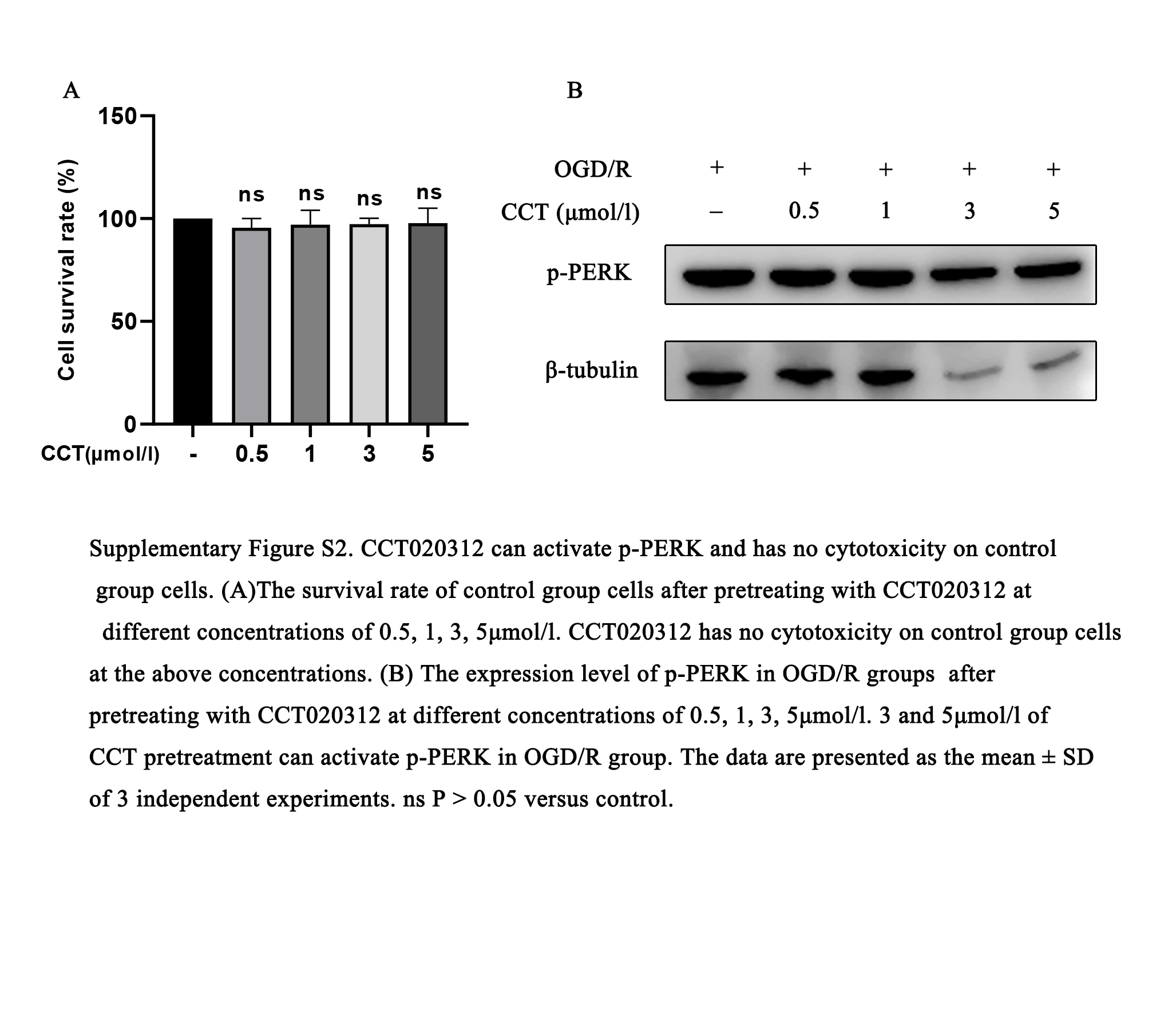

Supplement: Supplementary file 1 [file ijms-23-12983-s001.zip › Supplementary materials/Supplementary Figure S2.tif]

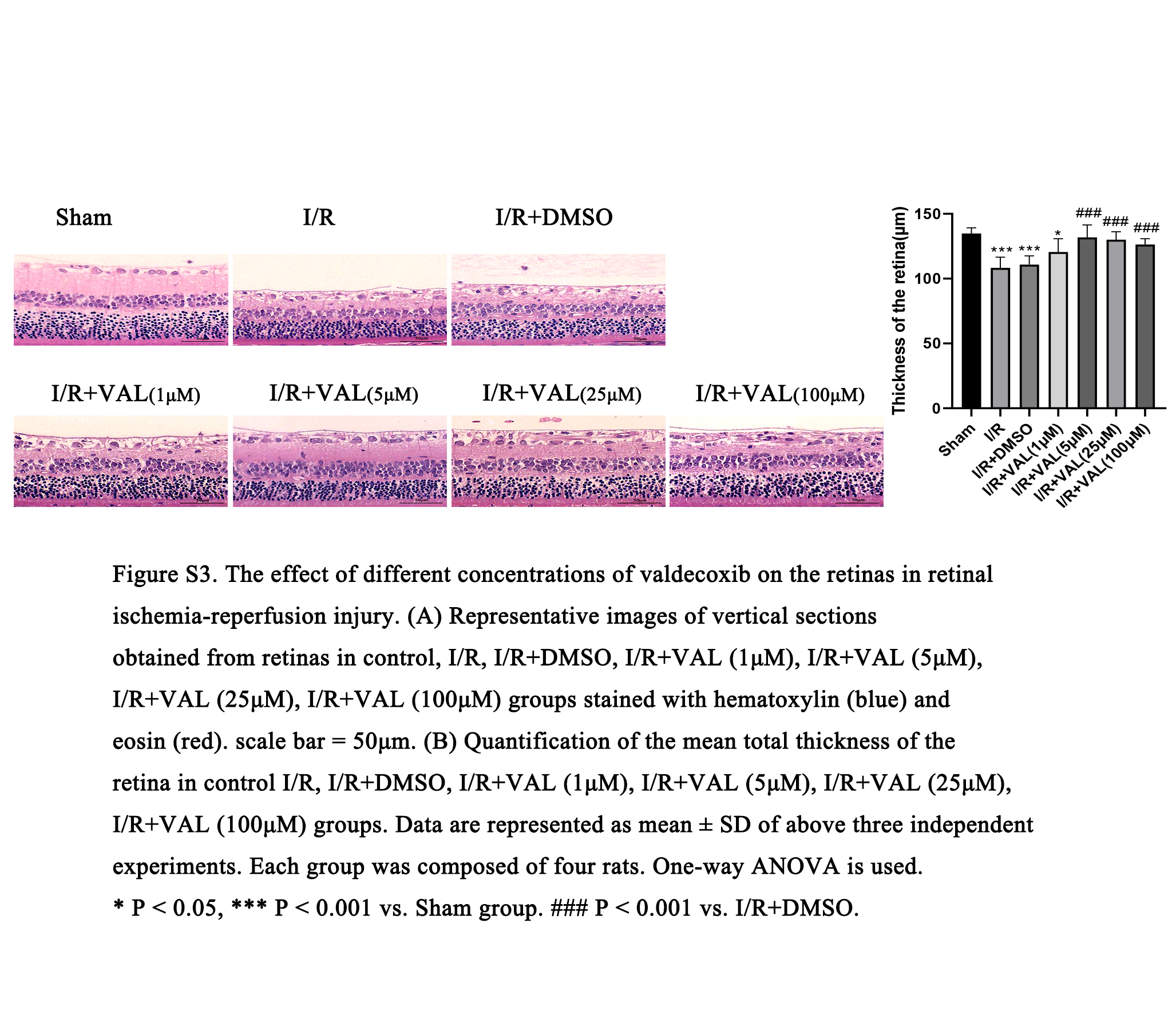

Supplement: Supplementary file 1 [file ijms-23-12983-s001.zip › Supplementary materials/Supplementary Figure S3.tif]
